# Selection and Validation of Reference Genes for Pan-Cancer in Platelets Based on RNA-Sequence Data

**DOI:** 10.3389/fgene.2022.913886

**Published:** 2022-06-13

**Authors:** Xiaoxia Wen, Guishu Yang, Yongcheng Dong, Liping Luo, Bangrong Cao, Birga Anteneh Mengesha, Ruiling Zu, Yulin Liao, Chang Liu, Shi Li, Yao Deng, Kaijiong Zhang, Xin Ma, Jian Huang, Dongsheng Wang, Keyan Zhao, Ping Leng, Huaichao Luo

**Affiliations:** ^1^ Chongqing Key Laboratory of Sichuan-Chongqing Co-construction for Diagnosis and Treatment of Infectious Diseases Integrated Traditional Chinese and Western Medicine, College of Medical Technology, Chengdu University of Traditional Chinese Medicine, Chengdu, China; ^2^ Department of Clinical Laboratory, Guangyuan Central Hospital, Guangyuan, China; ^3^ GenomCan Inc., Chengdu, China; ^4^ Sichuan Cancer Hospital and Institute, Sichuan Cancer Center, School of Medicine, University of Electronic Science and Technology of China, Chengdu, China; ^5^ Center for Informational Biology, School of Life Science and Technology, University of Electronic Science and Technology of China, Chengdu, China; ^6^ Department of Clinical Laboratory, Sichuan Cancer Hospital and Institute, Sichuan Cancer Center, School of Medicine, University of Electronic Science and Technology of China, Chengdu, China

**Keywords:** platelets, reference genes, quantitative real time polymerase chain reaction, normalization, pan-cancer

## Abstract

Many studies in recent years have demonstrated that some messenger RNA (mRNA) in platelets can be used as biomarkers for the diagnosis of pan-cancer. The quantitative real-time polymerase chain reaction (RT-qPCR) molecular technique is most commonly used to determine mRNA expression changes in platelets. Accurate and reliable relative RT-qPCR is highly dependent on reliable reference genes. However, there is no study to validate the reference gene in platelets for pan-cancer. Given that the expression of some commonly used reference genes is altered in certain conditions, selecting and verifying the most suitable reference gene for pan-cancer in platelets is necessary to diagnose early stage cancer. This study performed bioinformatics and functional analysis from the RNA-seq of platelets data set (GSE68086). We generated 95 candidate reference genes after the primary bioinformatics step. Seven reference genes (*YWHAZ*, *GNAS*, *GAPDH*, *OAZ1*, *PTMA*, *B2M*, and *ACTB*) were screened out among the 95 candidate reference genes from the data set of the platelets’ transcriptome of pan-cancer and 73 commonly known reference genes. These candidate reference genes were verified by another platelets expression data set (GSE89843). Then, we used RT-qPCR to confirm the expression levels of these seven genes in pan-cancer patients and healthy individuals. These RT-qPCR results were analyzed using the internal stability analysis software programs (the comparative Delta CT method, geNorm, NormFinder, and BestKeeper) to rank the candidate genes in the order of decreasing stability. By contrast, the *GAPDH* gene was stably and constitutively expressed at high levels in all the tested samples. Therefore, *GAPDH* was recommended as the most suitable reference gene for platelet transcript analysis. In conclusion, our result may play an essential part in establishing a molecular diagnostic platform based on the platelets to diagnose pan-cancer.

## Introduction

Platelets are derived from the megakaryocytes of the bone marrow, which are abundant in the peripheral blood (Jain et al., 2013). Platelets have long been considered to only stimulate coagulation after tissue trauma or vascular injury (Holinstat, 2017; Roweth and Battinelli, 2021). However, recent studies have shown that platelets are involved in multiple stages of cancer and are potential cancer diagnostic biomarkers (Zu et al., 2020). In the past, it was believed that the platelet content was static because platelets are cell fragments lacking a nucleus, and therefore no transcription and translation were expected (‘t Veld and Wurdinger, 2019) until some researchers demonstrated that platelets have the ability for protein synthesis (Warshaw et al., 1966; Burkhart et al., 2012), and the mRNA is involved in the protein synthesis reaction in platelets (Harrison and Goodall, 2008). It has been well appreciated that platelets can obtain a diverse range of mRNAs from megakaryocytes, translating into protein under external stimuli (Raslova et al., 2007). Studies have proved that tumor cells can directly stimulate platelet protein synthesis, while platelets can also sequester tumor-associated biomolecules such as proteins and RNA (‘t Veld and Wurdinger, 2019; Klement et al., 2009; Nilsson et al., 2016). The combination of specific splicing events in response to external signals and the ability of platelets to directly splice the circulating mRNA provides a highly dynamic transcriptome for platelets potentially suitable for liquid biopsies for cancer diagnosis (‘t Veld and Wurdinger, 2019; Harrison and Goodall, 2008; Best et al., 2018; Nassa et al., 2018). Given this situation, the concept of tumor-educated platelets (TEPs) has been proposed in recent years, referring to those platelets that can interact with the tumor cells and change the RNA profile (Best et al., 2015). TEP mRNAs have been confirmed to be dynamically affected by tumor conditions and may serve as biomarkers for cancer diagnosis, prognosis, prediction, or monitoring (Xue et al., 2018; Wurdinger et al., 2020).

RT-qPCR has been considered a sensitive, efficient, and reliable molecular technique to determine the mRNA levels (Xiong et al., 2018; Lin et al., 2019). Studies have proved that RT-qPCR can also amplify platelet-derived mRNA even though the concentration of mRNA is low in the whole platelets (Newman et al., 1988). Accurate and reliable relative RT-qPCR is highly dependent on reliable reference genes (Deng et al., 2020). The use of inappropriate reference genes can result in incorrect findings (Zhou et al., 2018). Therefore, the selection of reference genes depends on various species and under different experimental conditions (Coulson et al., 2008; Wang et al., 2021). However, the reference genes in the current studies of differential gene expression between the platelets and different cancers have not been uniform (Table 1). Most reference genes in platelets were found to directly use tissues' or cells’ reference genes. Different reference genes were also used in the same cancer study. Firstly, we cannot determine whether the reference genes of cells and tissues can be applied to platelets. Furthermore, it is unclear whether the most appropriate selection of reference genes in platelets will differ due to the different cancers. Therefore, selecting and verifying the most suitable reference gene for pan-cancer in platelets is necessary.

**TABLE 1:** An overview of the current reference genes commonly used for studies of pan-cancer and platelets.

References gene	Cancer type	Sample size	PMID
*GAPDH*	Colorectal cancer (CRC)	286 CRC patients and 41 healthy controls and 22 patients with ulcerative colitis and 23 patients with Crohn’s disease	31639773
*ACTB*	Lung cancer	48 lung cancer patients and 48 healthy donors	31552488
*GAPDH*	Colorectal cancer (CRC) and non–small-cell lung cancer (NSCLC)	19 CRC patients, 16 NSCLC patients, and 4 healthy volunteers	33955587
*ACTB*	Non–small-cell lung cancer (NSCLC)	243 NSCLC patients, 150 healthy controls, and 141 benign pulmonary nodules patients	31523198
*ACTB* and *GAPDH*	Non–small-cell lung cancer (NSCLC)	10 NSCLC patients and 7 healthy subjects	33287695
*ACTB*	Hepatocellular carcinoma (HCC)	20 HCC patients, 20 liver cirrhosis patients, and 10 healthy subjects	34469466
*ACTB*	Lung cancer	58 healthy donors and 156 lung cancer patients	30201066

This study is aimed to screen out the candidate genes expressed stably through the platelets’ transcript data set analysis and verify their expression stability in the platelets of pan-cancer patients by the RT-qPCR method. Then, the computer program Delta CT method (Silver et al., 2006), BestKeeper (Pfaffl et al., 2004), geNorm (Vandesompele et al., 2002), and NormFinder (Andersen et al., 2004) were used for a comprehensive analysis of the expression stability of the candidate genes. The reference gene, expressed most stably in the platelets, can be used as an internal control for the quantitative gene assay. It will promote establishing a molecular diagnostic platform based on the TEPs, to diagnose and monitor pan-cancer.

## Materials and Methods

### Data Collection and Bioinformatics Analysis

We used data set GSE68086, the RNA-sequencing data of platelets, with six different malignant tumors (non–small-cell lung cancer, colorectal cancer, pancreatic cancer, glioblastoma, breast cancer, and hepatobiliary carcinomas). It is available in the public repository of the Gene Expression Omnibus (GEO) database supported by the National Center for Biotechnology Information (NCBI) (Best et al., 2015). For further downstream analyses, the reads were quality controlled using Trimmomatic (Bolger et al., 2014), mapped to the human reference genome using STAR (Dobin et al., 2013), and intron-spanning reads were summarized using HTseq (Anders et al., 2015). The processed data include 285 samples (columns) and 57,736 ensemble gene ids (rows). Firstly, the samples that yielded less than 0.4 × 10^6 intron-spanning reads were excluded. Genes with a count of 0 in more than 70% of the total sample size were also deleted. Besides, genes were further excluded by following the three filtration criteria (Cheng et al., 2011; Maltseva et al., 2013; Zhan et al., 2014) for being highly and stably expressed in platelets across normal and tumor samples.

1) Mean (normal)/mean (tumor) < 1.2 and mean (tumor)/mean (normal) < 1.2. The mean of the log2*CPM* value of the mRNA in normal and tumor samples. We retained the positive and negative 1.2-fold genes in the normal and tumor samples.
2) Top 10% mean normal and top 10% mean tumor samples were included. We retained the first 10% of genes in the normal and tumor samples.
3) CV (coefficient of variation) (normal) <10% and CV (tumor) <10%. We retained genes with CV <10% in the tumor and normal samples. CV = standard deviation (SD)/mean.

### Participants in the Validation Group

The tumor participants were included as follows: 1) patients with clinically suspected cancer were admitted to the Sichuan Cancer Hospital based on the guidelines; 2) patients without preoperative chemotherapy or radiotherapy; and 3) all the final diagnoses were based on pathology examinations. The tumor patients were excluded as follows: 1) patients with a previous history of antiplatelet medications such as aspirin; 2) pregnant patients; 3) patients with infections; and 4) patients without comprehensive clinical information. All the healthy participants were included with no disease. This study was approved by the medical ethical committee of the Sichuan Cancer Hospital (SCCHEC-02-2020-043).

### Platelet Isolation

The blood samples of all tumor participants were collected preoperatively. 1.5 ml of EDTA anticoagulated blood was added to 2 ml of EP tube. Platelet-rich plasma (PRP) was separated from the nucleated blood cells by a 20-min 120 × *g* centrifugation step using the centrifuge (Shuke Instrument, Sichuan, China), while the platelets were separated from PRP by centrifuging at 360 × *g* for 20 min. To minimize the impact of time, the isolation was supposed to be completed within 2 h after blood collection.

### RNA Isolation and cDNA Synthesis

Total RNA was extracted from the platelets using a TRIzol reagent (Ambion, United States). The concentration and quality of the total RNA were assessed using Thermo Scientific NanoDrop 2000 Spectrophotometer (Thermo Scientific, United States). Reverse transcription was performed using a PrimeScript RT reagent kit with a gDNA eraser (TaKaRa Bio, Dalian, China) following the manufacturer’s instructions.

### Quantitative Real-Time Polymerase Chain Reaction

The primers used in the study are listed in Table 2. All the primers were designed and synthesized by Tsingke Biological Technology (Beijing, China). Quantitative real-time polymerase chain reaction (RT-qPCR) was carried out using the CFX Connect Real-Time PCR Detection System (Bio-Rad, Shanghai, China), in which the amplification and detection steps were combined. The reactions were performed using the TB Green Premix Ex Taq II PCR kit (TaKaRa; Dalian, China). All the assays were performed using three biological replicates. A single qPCR reaction was performed in a 20 µL volume containing 10 µL SYBR Green Master Mix, 0.8 µL of each primer, 2 µL of cDNA sample, and 6.4 µL water free of RNase and DNase.

**TABLE 2:** Primer sequences of the seven candidate reference genes.

Gene	Primer sequences (5′–3′)
*ACTB*	F:GCTATACGACCTGCTGCCTTTCT
	RR:CTC​CTT​AAT​GTC​ACG​CAC​GAT
	CTCCTTAATGTCACGCACGAT
*GAPDH*	F:ACCCAGAAGACTGTGGATGG
	R:TTCAGCTCAGGGATGACCTT
*YWHAZ*	F:CCTGCATGAAGTCTGTAACTGAG
	R:GACCTACGGGCTCCTACAACA
*B2M*	F:GAGGCTATCCAGCGTACTCCA
	R:CGGCAGGCATACTCATCTTTT
*GNAS*	F:TGCCTCGGGAACAGTAAGAC
	R:GCCGCCCTCTCCATTAAAC
*OAZ1*	F:CTCCACTGCTGTAGTAACCCG
	R:GATCCCTCTGACTATTCCCTCG
*PTMA*	F:TCAGACGCAGCCGTAGACA
	R:GCATTCCCGTTAGCAGGGG

### Stability Assessment of Candidate Genes

The mRNAs with a cycle threshold (Ct) value less than 35 in the panel were included in the data analysis. The average expression stability of the candidate reference genes was also evaluated by the computer programs Delta CT method, BestKeeper, geNorm, and NormFinder. The Delta CT algorithm calculated δCt by comparing the relative expression of “gene pairs” in each sample, used as a criterion for screening the reference genes. The BestKeeper algorithm calculated the correlation coefficient r, SD, and CV of the gene pairing to screen out the most stable expression reference gene. The geNorm algorithm mainly evaluated the stability of candidate gene expression by testing the stability M value and average pairwise variation (V) of the algorithm to screen out more than one reference gene. The parameter calculated by the NormFinder was the stability value, which is related to the systematic error of each candidate gene.

Then, the reference gene that we finally screened out was analyzed for stability of its expression in various cancers *via* the Platelet Expression Atlas website (http://bioinfo.life.hust.edu.cn/PEA/#!/). This is a comprehensive platelet expression atlas (PEA) resource and platelet transcriptome landscape website which collects platelet expression data sets, including 1260 RNA-seq, 358 RNA microarray, 21 miRNA-seq, and 430 miRNA microarray data sets from 27 disease types and healthy controls from the gene expression omnibus of the National Center For Biotechnology Information (NCBI GEO) and sequence read archive (SRA) databases (Xie et al., 2022).

### Validation of Reference Gene

The reference gene we selected was further used to verify the differential gene expression between healthy subjects and lung cancer patients through RT-qPCR experiments to better evaluate its clinical application value as a reference gene. Statistical analysis was performed with GraphPad 8.4. Student’s *t*-test or two-sided χ^2^ test was used to compare the differences in other variables among the groups. A *p* value < 0.05 was considered to be statistically significant.

## Results

### Shortlisting of Reference Genes

**FIGURE 1:**
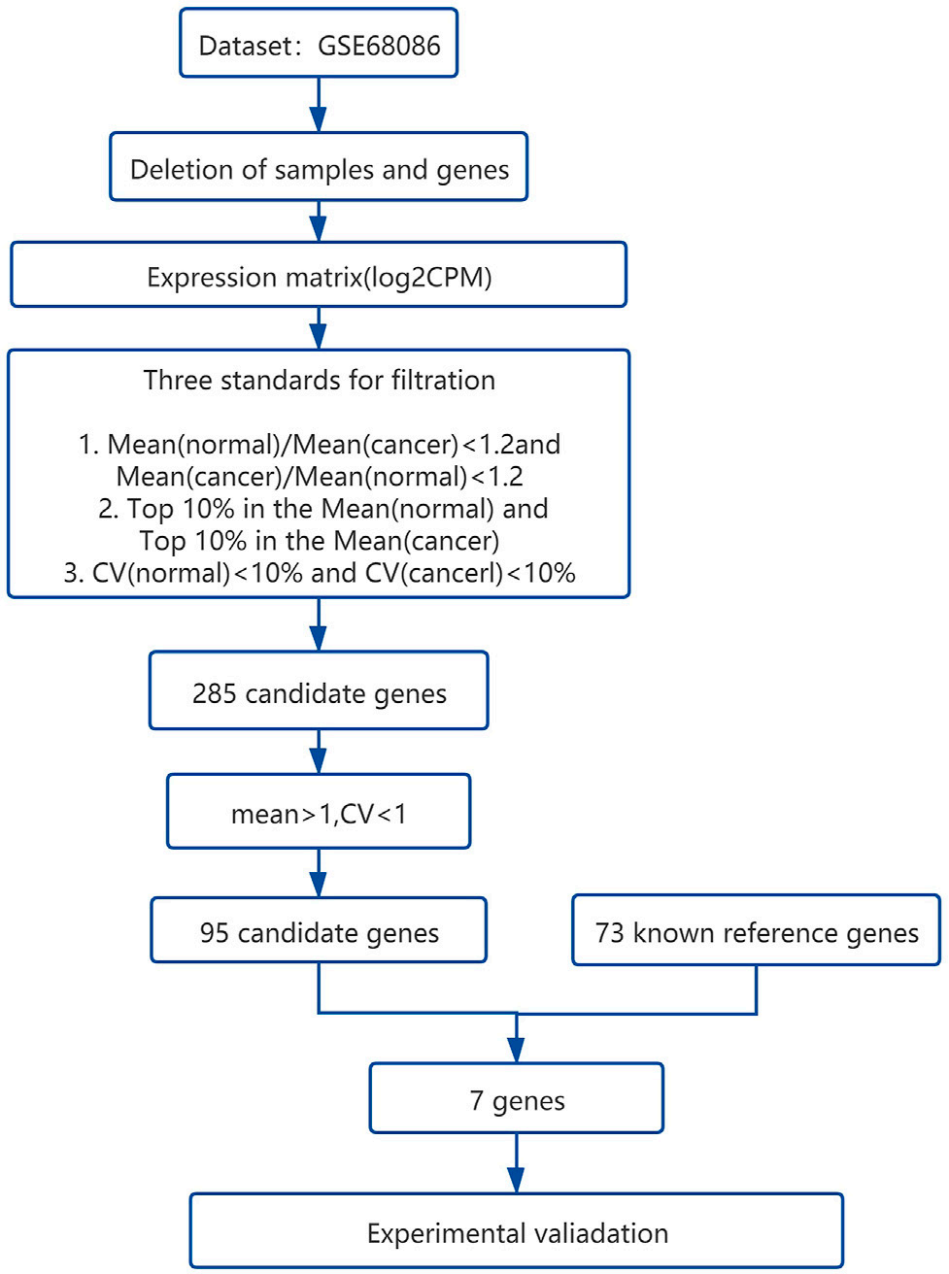
The overall workflow of bioinformatical statistics for screening the candidate reference genes from the platelet RNA sequencing data set.

A total of 285 candidate genes were obtained after processing the data set GSE68086. The overall workflow of the present study is shown in Figure 1, and the details are stated in the Materials and Methods section. We further have 95 candidate genes after optimizing the mean >1 and CV < 1 from the 285 of our pre-evaluation reference genes (Supplementary Table S1). After that, we compared 95 genes with 73 known reference genes (Radonić et al., 2004; Zhang et al., 2005; Tratwal et al., 2014; Ayakannu et al., 2015; Sharan et al., 2015; Walter et al., 2016; Panina et al., 2018; Zhao et al., 2018; Zhang et al., 2022) and finally got seven candidate genes (YWHAZ, GNAS, GAPDH, OAZ1, PTMA, B2M, and ACTB). These seven genes are known as the reference genes and are also stably expressed genes selected from the platelet data set.

**FIGURE 2:**
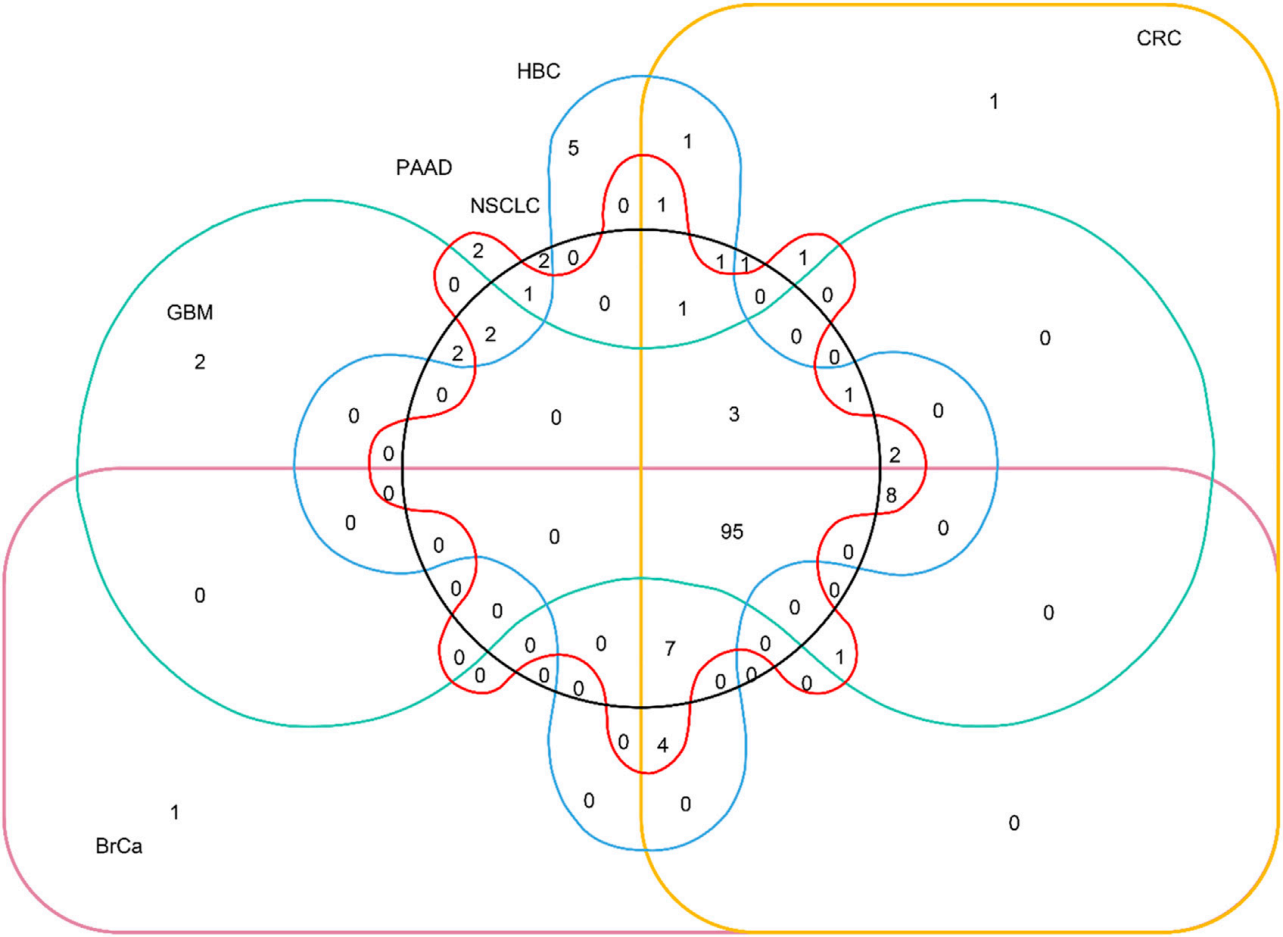
Venn diagram of distribution relationship between the candidate genes and the six tumor groups. The 95 genes in the lower right corner are selected from 285 candidate genes.

**FIGURE 3:**
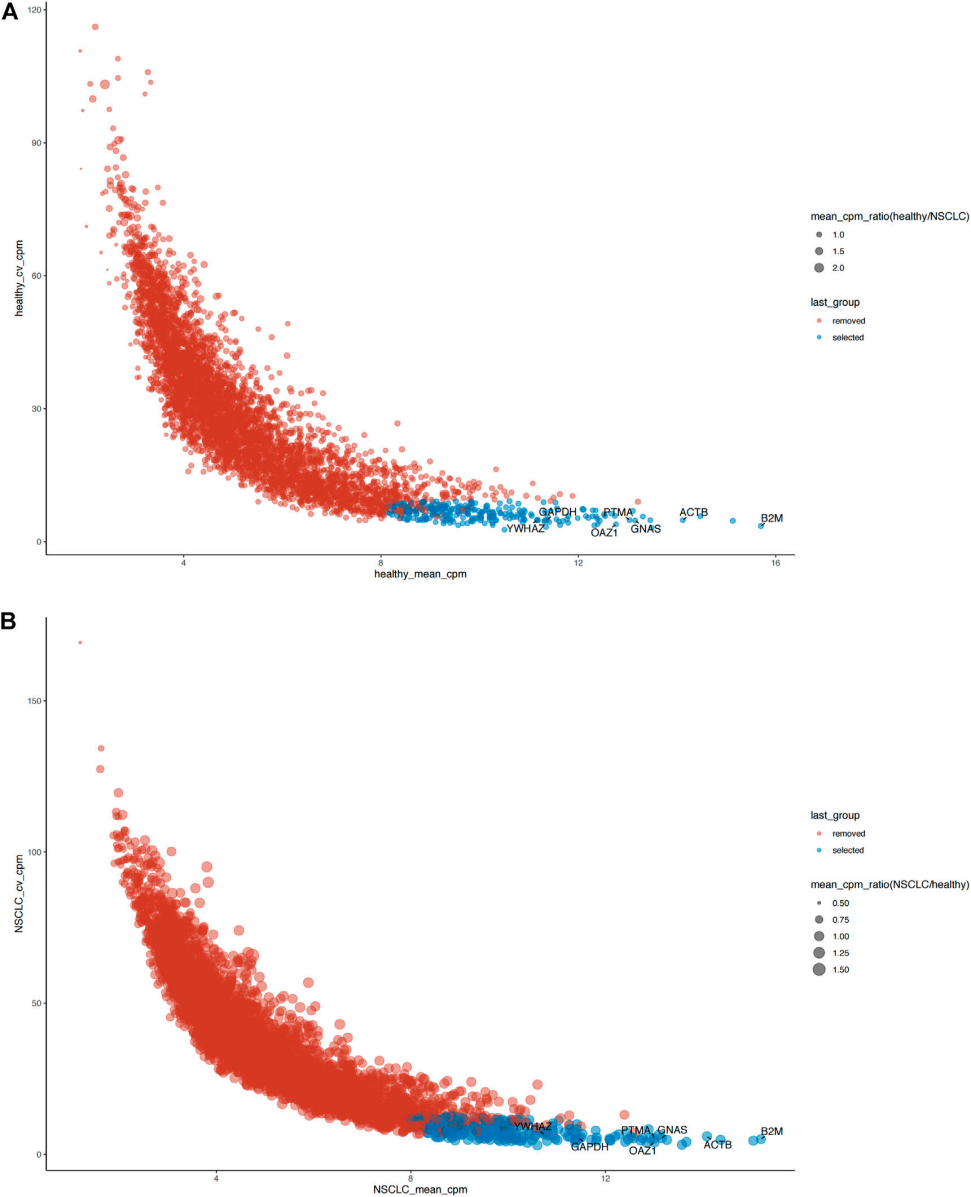
The verification of expression stability of seven candidate reference genes of platelet in another data set (GSE89843). **(A)** Seven candidate reference genes all expressed stably in the platelet sequencing data from 377 healthy individuals. **(B)** Seven candidate reference genes all expressed stably in the platelet sequencing data from 402 NSCLC patients. Blue: selected (stable expression), red: removed (unstable expression).

The distribution relationship between the candidate genes and the six tumor groups [glioblastoma (GBM), breast cancer (BrCa), pancreatic cancer (PAAD), non–small-cell lung cancer (NSCLC), hepatobiliary cancer (HBC), and colorectal cancer (CRC)] is shown in Figure 2. We then used the same bioinformatics analysis conditions (Materials and Methods section) of data set GSE68086 to analyze data set GSE89843, to verify the stability of the seven candidate genes we selected. The data set GSE89843 consists of 402 platelet samples from NSCLC patients in different stages and 377 from the healthy subjects. The result show that all the seven candidate genes that we selected also expressed stably in another platelet data set (GSE89843) (Figure 3).

### Stability Assessment of Seven Candidate Reference Genes

**FIGURE 4:**
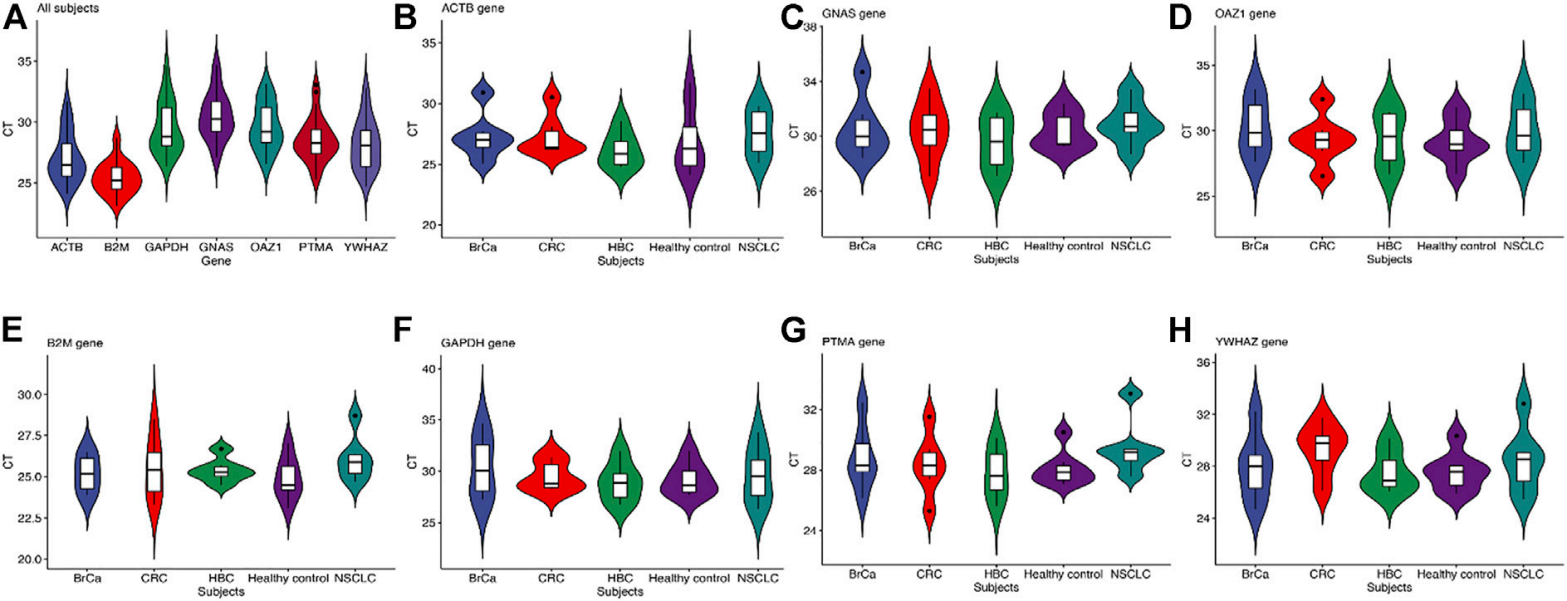
**(A)** Validation of the stability of the seven reference genes. **(B-H)** The validation of each reference gene with the cancerous and health group. CT value reflects the abundance of reference gene expression. The higher the CT value, the lower the expression level, and *vice versa*. The standard deviation (SD) of CT values is a schematic indicator of the stability of candidate reference gene expression in all the tested samples. The box plots show a box from the first quartile (25th percentiles) to the third quartile (75th percentiles) and the median in the midst (50th percentiles).

The baseline characteristics of all the participants are listed in Table 3. A total of 30 subjects were included in the first validation step: non–small-cell lung carcinoma (NSCLC, n = 5), colorectal cancer (CRC, n = 6), hepatobiliary cancer (HBC, n = 6), breast cancer (BrCa, n = 6), and healthy subjects (HC, n = 7). The results are shown in Figure 4. All statistical measures have been primarily done on each of the seven reference genes in all the 30 subjects (Figure 4A). Then, all the measurements were calculated for all the reference genes in a specific tumor and healthy group (Supplementary Table S2). The reference gene *B2M* was highly stable and more expressed, scoring a mean = 25.40, median = 25.19, and SD = 1.33 (Supplementary Table S3). We also found that *B2M* was more stable in hepatobiliary and breast cancers than the other genes. The expressions of *GAPDH* in colon cancer, *PTMA* in healthy control, and *GNAS* in non–small-cell lung cancer were also higher (Figure 4B–H). The overall results indicated that *GAPDH*, *B2M*, and *ACTB* were more stable in each cancer than the other four candidate genes.

**TABLE 3:** Baseline characteristics of all the enrolled subjects.

	Total	Validation group 1	Validation group 2
Gender			
** **Male	37 (46.25%)	14 (46.7%)	23 (46%)
** **Female	43 (53.75%)	16 (53.3%)	27 (54%)
Age (years)			
Mean (SD)	57.46 (9.24)	60.47 (8.50)	55.6 (9.20)
Cancer			
Lung cancer (NSCLC)	15 (18.75%)	5 (16.67%)	10 (20.0%)
Colon cancer (CRC)	16 (20.0%)	6 (20.0%)	10 (20.0%)
Hepatobiliary cancer (HBC)	16 (20.0%)	6 (20.0%)	10 (20.0%)
Breast cancer (BrCa)	16 (20.0%)	6 (20.0%)	10 (20.0%)
Healthy control (HC)	17 (21.25%)	7 (23.33%)	10 (20.0%)
Stage			
I	3 (3.75%)	1 (3.3%)	2 (4.0%)
II	16 (20.0%)	7 (23.3%)	9 (18.0%)
III	13 (16.25%)	7 (23.3%)	6 (12.0%)
IV	27 (33.75%)	8 (26.7%)	19 (38.0%)
NA	21 (26.25%)	7 (23.3%)	14 (28.0%)

### Stability Assessment of *GAPDH*, *B2M*, and *ACTB*

**FIGURE 5:**
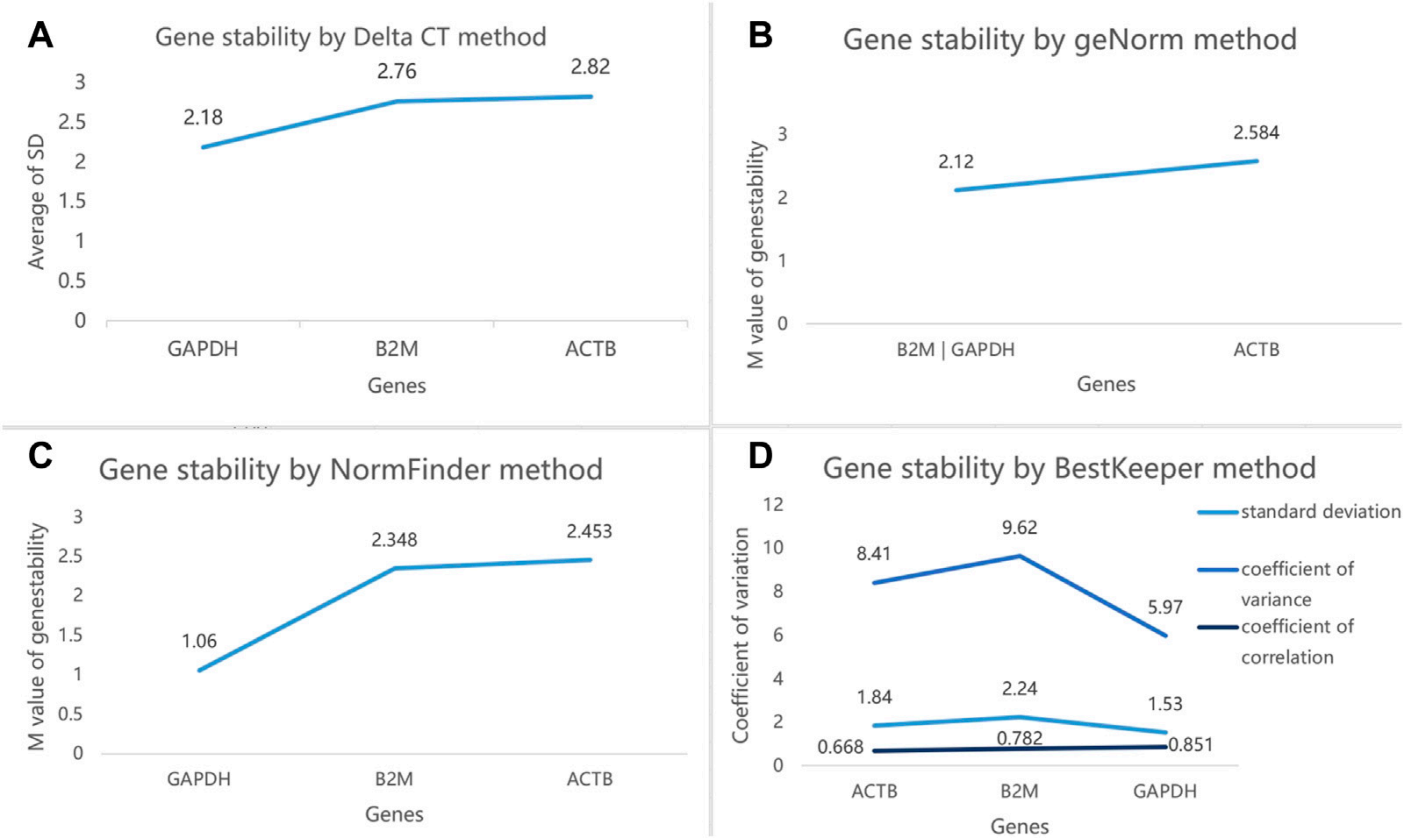
**(A)** Ranking of the three reference genes based on their expression stability calculated by Delta CT. **(B)**. GeNorm analysis of the three candidate reference genes. **(C)**. Stability value of each of the three candidate reference genes from the NormFinder analysis. **(D)**. BestKeeper algorithm analysis to determine the stability of reference genes, where a low value indicates a more stable expression in the normalization factor. The least stable gene in each step is indicated by arrows.

Then, we selected these three reference genes *GAPDH*, *B2M*, and *ACTB* with higher expression stability to validate in the second step. A total of 50 subjects were included in this step, that is, 10 NSCLC, 10 CRC, 10 HBC, 10 BrCa, and 10 HC (Table 3). The mean Ct values of the three reference genes in the 50 subjects are shown in Supplementary Table S4. The web-based four algorithms were applied (Xie et al., 2012) to compare stability among the three reference genes. The Delta CT method analyses were performed to rank the genes according to the overall stability across the 50 individuals (Figure5A). The average expression stability (M) value from the GeNorm analysis was lower than 2.7 for the most stable candidates. According to geNorm, *B2M* was highly expressed parallel to *GAPDH* (Figure 5B). The ranking of the genes in the NormFinder analysis was almost similar to the Delta CT ranking (Figure 5C). The BestKeeper algorithm calculated the correlation coefficient r, SD, and CV of the gene pairing, and the results showed that *GAPDH* is the most stably expressed reference gene (Figure 5D). Furthermore, the candidate reference genes were ranked in the increasing order of their stability values, and the *GAPDH* was the best reference gene in platelets for pan-cancer (Table 4).

**TABLE 4:** Ranking of the three reference genes stability.

Methods	References genes stability value rank		
	First (1st)	Second (2nd)	Third (3rd)
Delta CT	*GAPDH*	*B2M*	*ACTB*
BestKeeper	*GAPDH*	*ACTB*	*B2M*
NormFinder	*GAPDH*	*B2M*	*ACTB*
geNorm	*B2M/GAPDH*	—	*ACTB*
Recommended comprehensive ranking	***GAPDH***	***B2M***	***ACTB***

Bold values indicates that the finally comprehensive ranking of three reference genes after combing other stability methods.

Finally, according to the analysis results from the website of the Platelet Expression Atlas, the expression of *GAPDH* in platelets was stable in a variety of cancers when compared with that in healthy subjects, including some uncommon cancers. GAPDH in platelets were differentially expressed only in ST elevation myocardial infarction and HIV, dengue, and H1N1 (*p* < 0.05) (Supplementary Table S5). Therefore, the results further proved that *GAPDH* was suitable as a reference gene in the platelets for pan-cancer.

### Validation of the Clinical Application Value for *GAPDH* As a Reference Gene

The abovementioned analysis results showed that *GAPDH* was more suitable as a reference gene for pan-cancer platelet transcriptome quantitative analysis. To evaluate its clinical value as a reference gene more comprehensively, a new RT-qPCR experiment was designed. We selected the differential gene *FLNA*, which was significantly different in lung cancer patients when compared with healthy subjects by analysis *via* the Platelet Expression Atlas website, to verify the differential expression of the *FLNA* in the platelets of lung cancer and healthy subjects by using *GAPDH* as the reference gene.

As shown in Table 5, 42 subjects were enrolled in this step: Lung cancer (LC, n = 21) and healthy subjects (HC, n = 21). There was no statistical difference in gender between the two groups (*p* > 0.05), while the age between the groups was statistically different and the patients with lung cancer were significantly older than the healthy subjects (*p* < 0.05).

**TABLE 5:** Basic clinical characteristics of the verified subjects.

	Lung cancer	Healthy subjects	*p* value
**Total**	21	21	—
**Gender**	—	—	0.298
** **Male	14 (66.7%)	9 (42.9%)	—
** **Female	7 (33.3%)	12 (57.1%)	—
**Age**	61.90 (7.01)	49.48 (8.41)	*0.001*
Mean (SD)			

Italic values represents the statistically significant different of age between the healthy subjects and lung cancer patients.

**FIGURE 6:**
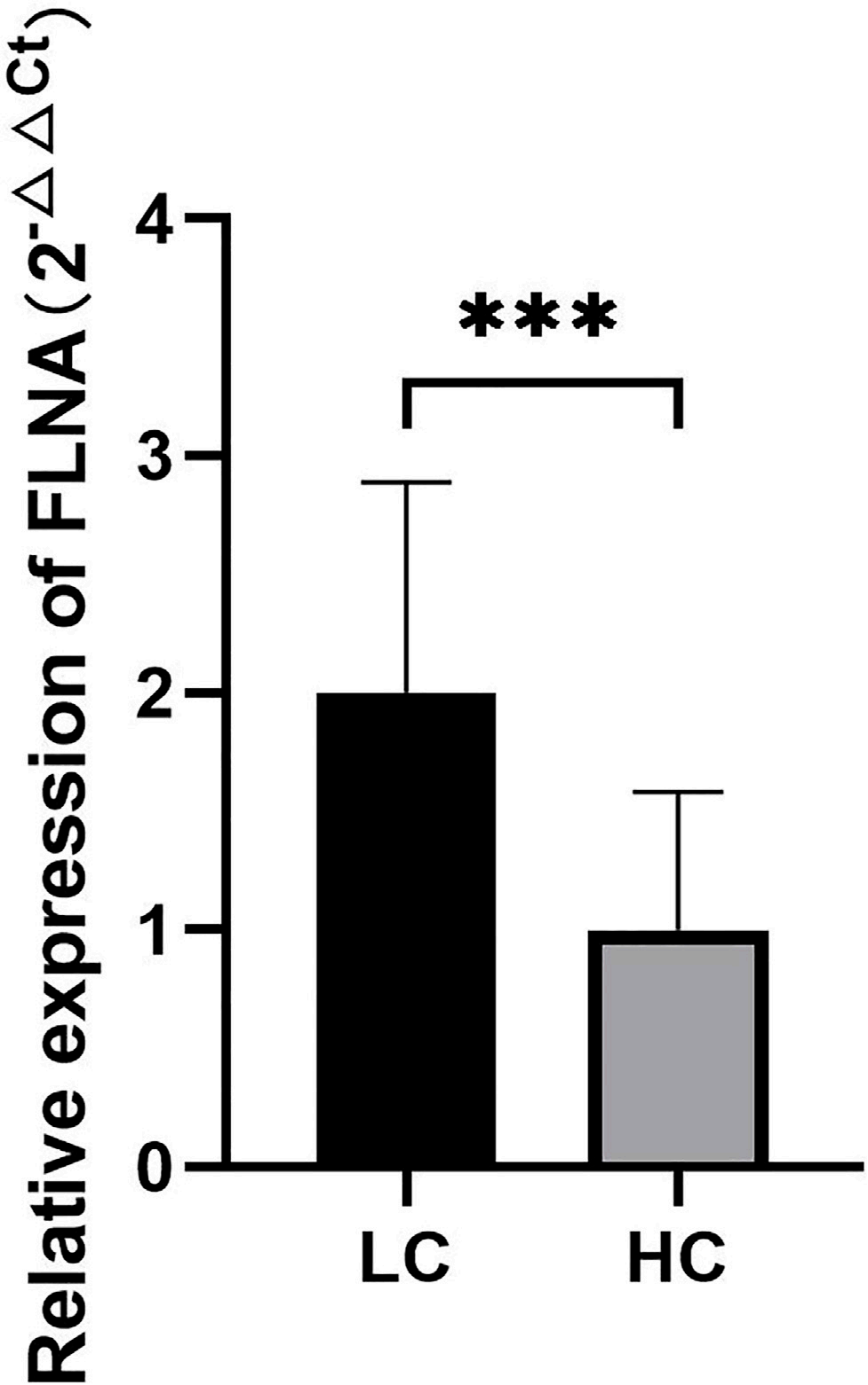
The quantitative analysis results of *FLNA* gene expression in platelets of lung cancer patients and healthy subjects. ***p < 0.01.

The results of RT-qPCR analysis showed that the expression of the *FLNA* gene in the two groups of platelets was statistically significant (*p* < 0.05) (Supplementary Table S6). The expression of *FLNA* was significantly higher in lung cancer patients than in normal people (Figure 6), indicating that *GAPDH* can be used as a reference gene for RT-qPCR analysis of tumor platelets, and also had a profound clinical application value for the early diagnosis of cancer.

## Discussion

Liquid biopsy technology based on blood biomarkers has developed rapidly in recent years, and various studies have shown that liquid biopsy is considered an important tool for early cancer detection (Chen and Zhao, 2019; Ignatiadis et al., 2021). Platelets are highly concerned as an emerging biological source of liquid biopsy (‘t Veld and Wurdinger, 2019). TEPs mRNA has been confirmed to be dynamically influenced by tumor conditions and may be used as a biomarker for many cancer diagnoses, prognosis, prediction, and monitoring (Best et al., 2015). There have been many studies on cancer detection and monitoring through differential expression of TEPs mRNA. Yang et al. (2019) used RT-qPCR to find significantly higher TEP *TIMP1* mRNA in colorectal cancer patients than in healthy individuals and in patients with ulcerative colitis and Crohn’s disease. Yao et al. (2019) found that the expression of TEP *TPM3* mRNA is significantly increased in BrCa patients, by using an RT-qPCR assay. Xing et al. (2019) proved that TEP *ITGA2B* mRNA expression is higher in NSCLC patients than in healthy individuals and in patients with benign lung nodules, by using RT-qPCR.

RT-qPCR is a technique with high sensitivity and specificity, which is widely applied in quantifying gene expression levels (Hellemans et al., 2007). It is important to set the reliable internal controls by the reference gene in the RT-qPCR quantification assay (Bustin et al., 2009). Many studies have shown that there is no single reference gene that could be effectively used in the RT-qPCR in all species or under all experimental conditions (Suzuki et al., 2000; Zhou et al., 2018). For example, Brzeszczyńska et al. (2020) proved that the classical reference gene in HepaRG cells such as *GAPDH* was altered by drug treatment. Vorachek et al. (2013) found that the commonly used reference genes, *PGK1*, *ACTB*, and *B2M* for neutrophils were not reliable reference genes under different conditions. The lack of gene expression stability makes it difficult to quantify and normalize RT-qPCR data. Therefore, reference genes with systematic identification and validation are essential for solving these problems. With more and more studies using TEPs mRNA for cancer detection and monitoring, it is urgent to screen out the stable reference genes in platelets for early cancer detection.

This study identified the stable reference gene in the platelets of pan-cancer patients and normal participants. In the past, there were few studies reported on the normalization of transcript levels for platelets. Two of the seven reference genes, *ACTB* and *GAPDH*, have been reported as normalization control in mRNA detection of RT-qPCR (Hurteau et al., 2006), which have also been confirmed to be expressed in neuroendocrine lung cancer (Walter et al., 2016). Our approach is different from the earlier study, in which the mRNAs were extracted and sequenced from the platelets. In addition, an analysis of platelets in patients with myocardial infarction showed that three reference genes, *HDGF*, *GNAS*, and *ACTB*, were reported as the most stable reference genes (Zsóri et al., 2013), while *ACTB* was one of the three most stable reference genes in our study. Another study on lung cancer cell division and platelets provided a potential platelet miRNA–based treatment strategy for lung cancer. It showed the importance of internal control in the detection of miRNA expression (Liang et al., 2015). But this study did not give a more detailed description of the selection of reference genes. Our research covered a wide range of cancers and selected the most suitable reference gene for platelet transcript research.

Surprisingly, our results revealed that reference genes’ stability and expressions varied from one cancer group to another. The *B2M* gene was expressed higher in hepatic carcinoma and breast cancer while being more stably expressed in liver cancer, and it has not yet been reported as per the knowledge we have. The *GAPDH* gene was more stable in colon cancer, and the *GNAS* gene was highly stable in lung cancer. Despite the possibility of different stable genes in various types of tumors, the overall reference genes validation indicated that *GAPDH*, *B2M*, and *ACTB* were the highly stable genes in the order of first to third consecutively in all the subjects. This study recommends *GAPDH* as a reference gene for pan-cancer normalization, providing a standard for quantitatively detecting the gene expression levels in platelets by using this reference gene as an internal control. We also suggested that further research has to be done on this reference gene with different systematic techniques on cancer-specific normalization for internal control.

There are some limitations to the present study that can be addressed in future work. On the one hand, the sample size and the type of cancer are not enough, which may introduce errors in this type of study. On the other hand, we only selected and validated the intersection among 95 candidate reference genes in the RNA-seq data set of the TEPs and 73 known reference genes. However, it is also necessary to consider choosing more specific platelet reference genes than the currently known reference genes for validation.

## Conclusion

In conclusion, we recommend *GAPDH* as the most suitable reference gene in platelets for pan-cancer normalization, providing a reference standard for quantitatively detecting the gene expression levels in platelets for the diagnosis of pan-cancer by using this reference gene as an internal control.

## Data Availability

The data sets presented in this study can be found in online repositories. The names of the repository/repositories and accession number(s) can be found in https://www.ncbi.nlm.nih.gov/, GSE68086, GSE89843.
