# Supplementary material for: Selection and Validation of Reference Genes for Pan-Cancer in Platelets Based on RNA-Sequence Data
Source: Front Genet. 2022 Jun 13;13:913886. doi: 10.3389/fgene.2022.913886 (PMC9234127; doi:10.3389/fgene.2022.913886)
Supplement: Supplementary file 1 [file DataSheet1.docx]

Supplementary Material

# Supplementary Table1. The descriptions of the 285 candidate reference genes.

| **Gene ID** | **Gene symbol** | **Nr-Annotation** |
| --- | --- | --- |
| ENSG00000185825 | BCAP31 | B-cell receptor-associated protein 31 |
| ENSG00000129562 | DAD1 | defender against cell death 1 |
| ENSG00000092199 | HNRNPC | heterogeneous nuclear ribonucleoprotein C |
| ENSG00000076944 | STXBP2 | syntaxin binding protein 2 |
| ENSG00000049323 | LTBP1 | latent transforming growth factor beta binding protein 1 |
| ENSG00000156265 | MAP3K7CL | MAP3K7 C-terminal like |
| ENSG00000162704 | ARPC5 | actin related protein 2/3 complex subunit 5 |
| ENSG00000198586 | TLK1 | tousled like kinase 1 |
| ENSG00000115956 | PLEK | pleckstrin |
| ENSG00000178057 | NDUFAF3 | NADH:ubiquinone oxidoreductase complex assembly factor 3 |
| ENSG00000064601 | CTSA | cathepsin A |
| ENSG00000138279 | ANXA7 | annexin A7 |
| ENSG00000143995 | MEIS1 | Meis homeobox 1 |
| ENSG00000250334 | LINC00989 | long intergenic non-protein coding RNA 989 |
| ENSG00000168497 | SDPR | serum deprivation response |
| ENSG00000101558 | VAPA | VAMP associated protein A |
| ENSG00000059377 | TBXAS1 | thromboxane A synthase 1 |
| ENSG00000130429 | ARPC1B | actin related protein 2/3 complex subunit 1B |
| ENSG00000127824 | tuba4a | tubulin alpha 4a |
| ENSG00000100225 | FBXO7 | F-box protein 7 |
| ENSG00000142669 | SH3BGRL3 | SH3 domain binding glutamate rich protein like 3 |
| ENSG00000184983 | NDUFA6 | NADH:ubiquinone oxidoreductase subunit A6 |
| ENSG00000070831 | CDC42 | cell division cycle 42 |
| ENSG00000124795 | DEK | DEK proto-oncogene |
| ENSG00000067560 | RHOA | ras homolog family member A |
| ENSG00000110934 | BIN2 | bridging integrator 2 |
| ENSG00000135218 | CD36 | CD36 molecule |
| ENSG00000075624 | ACTB | actin beta |
| ENSG00000111348 | ARHGDIB | Rho GDP dissociation inhibitor beta |
| ENSG00000072110 | ACTN1 | actinin alpha 1 |
| ENSG00000131778 | CHD1L | chromodomain helicase DNA binding protein 1 like |
| ENSG00000111229 | ARPC3 | actin related protein 2/3 complex subunit 3 |
| ENSG00000114978 | mob1a | MOB kinase activator 1A |
| ENSG00000118680 | MYL12B | myosin light chain 12B |
| ENSG00000002586 | CD99 | CD99 molecule |
| ENSG00000120885 | MIR6843 | microRNA 6843 |
| ENSG00000137076 | MIR6852 | microRNA 6852 |
| ENSG00000149600 | COMMD7 | COMM domain containing 7 |
| ENSG00000184178 | SCFD2 | sec1 family domain containing 2 |
| ENSG00000142657 | PGD | phosphogluconate dehydrogenase |
| ENSG00000101782 | RIOK3 | RIO kinase 3 |
| ENSG00000107438 | PDLIM1 | PDZ and LIM domain 1 |
| ENSG00000113558 | SKP1 | S-phase kinase associated protein 1 |
| ENSG00000120727 | PAIP2 | poly |
| ENSG00000075151 | EIF4G3 | eukaryotic translation initiation factor 4 gamma 3 |
| ENSG00000005961 | ITGA2B | integrin subunit alpha 2b |
| ENSG00000136754 | ABI1 | abl interactor 1 |
| ENSG00000166091 | CMTM5 | CKLF like MARVEL transmembrane domain containing 5 |
| ENSG00000089327 | FXYD5 | FXYD domain containing ion transport regulator 5 |
| ENSG00000117592 | PRDX6 | peroxiredoxin 6 |
| ENSG00000182220 | ATP6AP2 | ATPase H+ transporting accessory protein 2 |
| ENSG00000160310 | PRMT2 | protein arginine methyltransferase 2 |
| ENSG00000127920 | GNG11 | G protein subunit gamma 11 |
| ENSG00000162614 | NEXN | nexilin F-actin binding protein |
| ENSG00000134108 | ARL8B | ADP ribosylation factor like GTPase 8B |
| ENSG00000071127 | WDR1 | WD repeat domain 1 |
| ENSG00000108518 | PFN1 | profilin 1 |
| ENSG00000092841 | MYL6 | myosin light chain 6 |
| ENSG00000112335 | SNX3 | sorting nexin 3 |
| ENSG00000136404 | TM6SF1 | transmembrane 6 superfamily member 1 |
| ENSG00000081154 | PCNP | PEST proteolytic signal containing nuclear protein |
| ENSG00000143727 | ACP1 | acid phosphatase 1, soluble |
| ENSG00000102898 | NUTF2 | nuclear transport factor 2 |
| ENSG00000078668 | VDAC3 | voltage dependent anion channel 3 |
| ENSG00000164924 | YWHAZ | tyrosine 3-monooxygenase/tryptophan 5-monooxygenase activation protein zeta |
| ENSG00000189403 | HMGB1 | high mobility group box 1 |
| ENSG00000187109 | NAP1L1 | nucleosome assembly protein 1 like 1 |
| ENSG00000100351 | GRAP2 | GRB2-related adaptor protein 2 |
| ENSG00000021355 | SERPINB1 | serpin family B member 1 |
| ENSG00000177324 | BEND2 | BEN domain containing 2 |
| ENSG00000135404 | CD63 | CD63 molecule |
| ENSG00000131966 | ACTR10 | actin-related protein 10 homolog |
| ENSG00000130755 | GMFG | glia maturation factor gamma |
| ENSG00000243317 | C7orf73 | chromosome 7 open reading frame 73 |
| ENSG00000140319 | SRP14 | signal recognition particle 14 |
| ENSG00000113732 | ATP6V0E1 | ATPase H+ transporting V0 subunit e1 |
| ENSG00000213625 | LEPROT | leptin receptor overlapping transcript |
| ENSG00000113851 | CRBN | cereblon |
| ENSG00000104904 | OAZ1 | ornithine decarboxylase antizyme 1 |
| ENSG00000204592 | HLA-E | major histocompatibility complex, class I, E |
| ENSG00000168610 | STAT3 | signal transducer and activator of transcription 3 |
| ENSG00000005020 | SKAP2 | src kinase associated phosphoprotein 2 |
| ENSG00000153071 | DAB2 | DAB2, clathrin adaptor protein |
| ENSG00000100664 | EIF5 | eukaryotic translation initiation factor 5 |
| ENSG00000082074 | FYB | FYN binding protein |
| ENSG00000149932 | TMEM219 | transmembrane protein 219 |
| ENSG00000070756 | MIR7705 | microRNA 7705 |
| ENSG00000167645 | YIF1B | Yip1 interacting factor homolog B, membrane trafficking protein |
| ENSG00000087086 | FTL | ferritin light chain |
| ENSG00000145335 | snca | synuclein alpha |
| ENSG00000132824 | SERINC3 | serine incorporator 3 |
| ENSG00000153064 | BANK1 | B-cell scaffold protein with ankyrin repeats 1 |
| ENSG00000147065 | MSN | moesin |
| ENSG00000111669 | TPI1 | triosephosphate isomerase 1 |
| ENSG00000198258 | UBL5 | ubiquitin like 5 |
| ENSG00000110696 | C11orf58 | chromosome 11 open reading frame 58 |
| ENSG00000117523 | PRRC2C | proline rich coiled-coil 2C |
| ENSG00000133112 | TPT1 | tumor protein, translationally-controlled 1 |
| ENSG00000165233 | CARD19 | caspase recruitment domain family member 19 |
| ENSG00000088726 | TMEM40 | transmembrane protein 40 |
| ENSG00000149925 | ALDOA | aldolase, fructose-bisphosphate A |
| ENSG00000166681 | BEX3 | brain expressed X-linked 3 |
| ENSG00000086666 | ZFAND6 | zinc finger AN1-type containing 6 |
| ENSG00000131171 | SH3BGRL | SH3 domain binding glutamate rich protein like |
| ENSG00000165119 | HNRNPK | heterogeneous nuclear ribonucleoprotein K |
| ENSG00000163736 | PPBP | pro-platelet basic protein |
| ENSG00000126267 | Cox6b1 | cytochrome c oxidase subunit 6B1 |
| ENSG00000148834 | GSTO1 | glutathione S-transferase omega 1 |
| ENSG00000129657 | SEC14L1 | SEC14 like lipid binding 1 |
| ENSG00000125971 | DYNLRB1 | dynein light chain roadblock-type 1 |
| ENSG00000124172 | ATP5E | ATP synthase, H+ transporting, mitochondrial F1 complex, epsilon subunit |
| ENSG00000155115 | GTF3C6 | general transcription factor IIIC subunit 6 |
| ENSG00000158869 | FCER1G | Fc fragment of IgE receptor Ig |
| ENSG00000170271 | FAXDC2 | fatty acid hydroxylase domain containing 2 |
| ENSG00000163737 | PF4 | platelet factor 4 |
| ENSG00000173660 | Uqcrh | ubiquinol-cytochrome c reductase hinge protein |
| ENSG00000115935 | WIPF1 | WAS/WASL interacting protein family member 1 |
| ENSG00000198168 | SVIP | small VCP interacting protein |
| ENSG00000132471 | WBP2 | WW domain binding protein 2 |
| ENSG00000205542 | TMSB4X | thymosin beta 4, X-linked |
| ENSG00000198843 | SELENOT | selenoprotein T |
| ENSG00000131143 | Cox4I1 | cytochrome c oxidase subunit 4I1 |
| ENSG00000118855 | MFSD1 | major facilitator superfamily domain containing 1 |
| ENSG00000174748 | RPL15 | ribosomal protein L15 |
| ENSG00000143933 | CALM2 | calmodulin 2 |
| ENSG00000123405 | NFE2 | nuclear factor, erythroid 2 |
| ENSG00000188921 | HACD4 | 3-hydroxyacyl-CoA dehydratase 4 |
| ENSG00000149357 | LAMTOR1 | late endosomal/lysosomal adaptor, MAPK and MTOR activator 1 |
| ENSG00000180628 | PCGF5 | polycomb group ring finger 5 |
| ENSG00000223855 | HRAT92 | heart tissue-associated transcript 92 |
| ENSG00000148700 | ADD3 | adducin 3 |
| ENSG00000161203 | AP2M1 | adaptor related protein complex 2 mu 1 subunit |
| ENSG00000137818 | RPLP1 | ribosomal protein lateral stalk subunit P1 |
| ENSG00000108953 | YWHAE | tyrosine 3-monooxygenase/tryptophan 5-monooxygenase activation protein epsilon |
| ENSG00000068796 | KIF2A | kinesin family member 2A |
| ENSG00000067225 | PKM | pyruvate kinase, muscle |
| ENSG00000061918 | GUCY1B3 | guanylate cyclase 1 soluble subunit beta |
| ENSG00000108839 | ALOX12 | arachidonate 12-lipoxygenase, 12S type |
| ENSG00000213719 | CLIC1 | chloride intracellular channel 1 |
| ENSG00000197746 | PSAP | prosaposin |
| ENSG00000130066 | SAT1 | spermidine/spermine N1-acetyltransferase 1 |
| ENSG00000070190 | DAPP1 | dual adaptor of phosphotyrosine and 3-phosphoinositides 1 |
| ENSG00000112096 | LOC100129518 | uncharacterized LOC100129518 |
| ENSG00000125952 | MAX | MYC associated factor X |
| ENSG00000180879 | SSR4 | signal sequence receptor subunit 4 |
| ENSG00000122566 | HNRNPA2B1 | heterogeneous nuclear ribonucleoprotein A2/B1 |
| ENSG00000104231 | ZFAND1 | zinc finger AN1-type containing 1 |
| ENSG00000125037 | EMC3 | ER membrane protein complex subunit 3 |
| ENSG00000120265 | PCMT1 | protein-L-isoaspartate |
| ENSG00000157514 | TSC22D3 | TSC22 domain family member 3 |
| ENSG00000105993 | DNAJB6 | DnaJ heat shock protein family |
| ENSG00000074800 | ENO1 | enolase 1 |
| ENSG00000023697 | DERA | deoxyribose-phosphate aldolase |
| ENSG00000130830 | MPP1 | membrane palmitoylated protein 1 |
| ENSG00000150681 | RGS18 | regulator of G-protein signaling 18 |
| ENSG00000148484 | RSU1 | Ras suppressor protein 1 |
| ENSG00000152061 | RABGAP1L | RAB GTPase activating protein 1 like |
| ENSG00000147394 | ZNF185 | zinc finger protein 185 |
| ENSG00000065534 | MYLK | myosin light chain kinase |
| ENSG00000099785 | 2-Mar | membrane associated ring-CH-type finger 2 |
| ENSG00000181061 | HIGD1A | HIG1 hypoxia inducible domain family member 1A |
| ENSG00000197415 | VEPH1 | ventricular zone expressed PH domain containing 1 |
| ENSG00000134996 | OSTF1 | osteoclast stimulating factor 1 |
| ENSG00000139644 | TMBIM6 | transmembrane BAX inhibitor motif containing 6 |
| ENSG00000087206 | UIMC1 | ubiquitin interaction motif containing 1 |
| ENSG00000102804 | TSC22D1 | TSC22 domain family member 1 |
| ENSG00000119801 | YPEL5 | yippee like 5 |
| ENSG00000257923 | CUX1 | cut like homeobox 1 |
| ENSG00000100504 | PYGL | phosphorylase, glycogen, liver |
| ENSG00000160014 | CALM3 | calmodulin 3 |
| ENSG00000165702 | GFI1B | growth factor independent 1B transcriptional repressor |
| ENSG00000143353 | LYPLAL1 | lysophospholipase like 1 |
| ENSG00000133317 | LGALS12 | galectin 12 |
| ENSG00000126581 | BECN1 | beclin 1 |
| ENSG00000113924 | HGD | homogentisate 1,2-dioxygenase |
| ENSG00000067167 | TRAM1 | translocation associated membrane protein 1 |
| ENSG00000048740 | CELF2 | CUGBP, Elav-like family member 2 |
| ENSG00000204264 | PSMB8 | proteasome subunit beta 8 |
| ENSG00000134882 | UBAC2 | UBA domain containing 2 |
| ENSG00000158710 | TAGLN2 | transgelin 2 |
| ENSG00000169756 | LIMS1 | LIM zinc finger domain containing 1 |
| ENSG00000010278 | CD9 | CD9 molecule |
| ENSG00000122545 | 7-Sep | septin 7 |
| ENSG00000221983 | UBA52 | ubiquitin A-52 residue ribosomal protein fusion product 1 |
| ENSG00000185787 | MORF4L1 | mortality factor 4 like 1 |
| ENSG00000149564 | ESAM | endothelial cell adhesion molecule |
| ENSG00000089053 | ANAPC5 | anaphase promoting complex subunit 5 |
| ENSG00000127314 | RAP1B | RAP1B, member of RAS oncogene family |
| ENSG00000161911 | TREML1 | triggering receptor expressed on myeloid cells like 1 |
| ENSG00000160948 | VPS28 | VPS28, ESCRT-I subunit |
| ENSG00000072042 | RDH11 | retinol dehydrogenase 11 |
| ENSG00000131100 | ATP6V1E1 | ATPase H+ transporting V1 subunit E1 |
| ENSG00000006652 | IFRD1 | interferon related developmental regulator 1 |
| ENSG00000134291 | TMEM106C | transmembrane protein 106C |
| ENSG00000167468 | GPX4 | glutathione peroxidase 4 |
| ENSG00000101856 | PGRMC1 | progesterone receptor membrane component 1 |
| ENSG00000148908 | RGS10 | regulator of G-protein signaling 10 |
| ENSG00000167461 | RAB8A | RAB8A, member RAS oncogene family |
| ENSG00000187514 | MIR1244-1 | microRNA 1244-1 |
| ENSG00000109332 | UBE2D3 | ubiquitin conjugating enzyme E2 D3 |
| ENSG00000148346 | LCN2 | lipocalin 2 |
| ENSG00000120690 | ELF1 | E74 like ETS transcription factor 1 |
| ENSG00000126432 | PRDX5 | peroxiredoxin 5 |
| ENSG00000184007 | PTP4A2 | protein tyrosine phosphatase type IVA, member 2 |
| ENSG00000184009 | ACTG1 | actin gamma 1 |
| ENSG00000102144 | PGK1 | phosphoglycerate kinase 1 |
| ENSG00000102265 | TIMP1 | TIMP metallopeptidase inhibitor 1 |
| ENSG00000257267 | ZNF271P | zinc finger protein 271, pseudogene |
| ENSG00000101162 | TUBB1 | tubulin beta 1 class VI |
| ENSG00000177156 | TALDO1 | transaldolase 1 |
| ENSG00000131236 | CAP1 | adenylate cyclase associated protein 1 |
| ENSG00000105402 | NAPA | NSF attachment protein alpha |
| ENSG00000105887 | MTPN | myotrophin |
| ENSG00000111644 | ACRBP | acrosin binding protein |
| ENSG00000149781 | FERMT3 | fermitin family member 3 |
| ENSG00000111640 | GAPDH | glyceraldehyde-3-phosphate dehydrogenase |
| ENSG00000136003 | ISCU | iron-sulfur cluster assembly enzyme |
| ENSG00000159346 | ADIPOR1 | adiponectin receptor 1 |
| ENSG00000174444 | RPL4 | ribosomal protein L4 |
| ENSG00000159348 | CYB5R1 | cytochrome b5 reductase 1 |
| ENSG00000137575 | SDCBP | syndecan binding protein |
| ENSG00000113140 | SPARC | secreted protein acidic and cysteine rich |
| ENSG00000101608 | MYL12A | myosin light chain 12A |
| ENSG00000088986 | DYNLL1 | dynein light chain LC8-type 1 |
| ENSG00000166946 | CCNDBP1 | cyclin D1 binding protein 1 |
| ENSG00000106052 | TAX1BP1 | Tax1 binding protein 1 |
| ENSG00000187742 | SECISBP2 | SECIS binding protein 2 |
| ENSG00000122643 | NT5C3A | 5'-nucleotidase, cytosolic IIIA |
| ENSG00000141027 | NCOR1 | nuclear receptor corepressor 1 |
| ENSG00000125356 | NDUFA1 | NADH:ubiquinone oxidoreductase subunit A1 |
| ENSG00000183726 | TMEM50A | transmembrane protein 50A |
| ENSG00000111716 | LDHB | lactate dehydrogenase B |
| ENSG00000180573 | Hist1h2ac | histone cluster 1 H2A family member c |
| ENSG00000155099 | TMEM55A | transmembrane protein 55A |
| ENSG00000135926 | MIR6513 | microRNA 6513 |
| ENSG00000095303 | PTGS1 | prostaglandin-endoperoxide synthase 1 |
| ENSG00000198668 | CALM1 | calmodulin 1 |
| ENSG00000138758 | 11-Sep | septin 11 |
| ENSG00000119684 | MLH3 | mutL homolog 3 |
| ENSG00000116288 | PARK7 | Parkinsonism associated deglycase |
| ENSG00000171735 | CAMTA1 | calmodulin binding transcription activator 1 |
| ENSG00000121766 | ZCCHC17 | zinc finger CCHC-type containing 17 |
| ENSG00000164096 | C4orf3 | chromosome 4 open reading frame 3 |
| ENSG00000100387 | RBX1 | ring-box 1 |
| ENSG00000166710 | B2M | beta-2-microglobulin |
| ENSG00000034713 | GABARAPL2 | GABA type A receptor associated protein like 2 |
| ENSG00000041353 | RAB27B | RAB27B, member RAS oncogene family |
| ENSG00000198898 | CAPZA2 | capping actin protein of muscle Z-line alpha subunit 2 |
| ENSG00000173812 | EIF1 | eukaryotic translation initiation factor 1 |
| ENSG00000085733 | CTTN | cortactin |
| ENSG00000183283 | DAZAP2 | DAZ associated protein 2 |
| ENSG00000143549 | TPM3 | tropomyosin 3 |
| ENSG00000103769 | RAB11A | RAB11A, member RAS oncogene family |
| ENSG00000102316 | MAGED2 | MAGE family member D2 |
| ENSG00000146376 | arhgap18 | Rho GTPase activating protein 18 |
| ENSG00000122862 | SRGN | serglycin |
| ENSG00000104763 | ASAH1 | N-acylsphingosine amidohydrolase 1 |
| ENSG00000196262 | PPIA | peptidylprolyl isomerase A |
| ENSG00000104765 | BNIP3L | BCL2 interacting protein 3 like |
| ENSG00000143409 | FAM63A | family with sequence similarity 63 member A |
| ENSG00000175324 | LSM1 | LSM1 homolog, mRNA degradation associated |
| ENSG00000115652 | UXS1 | UDP-glucuronate decarboxylase 1 |
| ENSG00000254999 | BRK1 | BRICK1, SCAR/WAVE actin nucleating complex subunit |
| ENSG00000197903 | Hist1h2bk | histone cluster 1 H2B family member k |
| ENSG00000117155 | SSX2IP | SSX family member 2 interacting protein |
| ENSG00000144746 | ARL6IP5 | ADP ribosylation factor like GTPase 6 interacting protein 5 |
| ENSG00000140941 | MAP1LC3B | microtubule associated protein 1 light chain 3 beta |
| ENSG00000163466 | ARPC2 | actin related protein 2/3 complex subunit 2 |
| ENSG00000168118 | RAB4A | RAB4A, member RAS oncogene family |
| ENSG00000163346 | PBXIP1 | PBX homeobox interacting protein 1 |
| ENSG00000087460 | GNAS | GNAS complex locus |
| ENSG00000151023 | ENKUR | enkurin, TRPC channel interacting protein |
| ENSG00000077549 | CAPZB | capping actin protein of muscle Z-line beta subunit |
| ENSG00000228474 | OST4 | oligosaccharyltransferase complex subunit 4, non-catalytic |
| ENSG00000140374 | ETFA | electron transfer flavoprotein alpha subunit |
| ENSG00000145703 | IQGAP2 | IQ motif containing GTPase activating protein 2 |
| ENSG00000189043 | NDUFA4 | NDUFA4, mitochondrial complex associated |
| ENSG00000168002 | POLR2G | RNA polymerase II subunit G |
| ENSG00000116171 | SCP2 | sterol carrier protein 2 |
| ENSG00000125676 | THOC2 | THO complex 2 |
| ENSG00000203879 | GDI1 | GDP dissociation inhibitor 1 |
| ENSG00000143761 | MIR3620 | microRNA 3620 |
| ENSG00000138293 | NCOA4 | nuclear receptor coactivator 4 |
| ENSG00000161570 | CCL5 | C-C motif chemokine ligand 5 |
| ENSG00000187667 | Unknown |  |

# Supplementary Table2. The Ct values of the seven candidate reference genes in the 30 subjects.

|  | ACTB | B2M | GAPDH | GNAS | OAZ1 | PTMA | YWHAZ |
| --- | --- | --- | --- | --- | --- | --- | --- |
| NSCLC1 | 26.79 | 25.07 | 28.03 | 30.90 | 27.54 | 29.04 | 28.28 |
| NSCLC2 | 25.85 | 26.38 | 26.35 | 31.96 | 32.19 | 29.39 | 26.36 |
| NSCLC3 | 25.14 | 25.63 | 27.49 | 28.73 | 28.22 | 27.59 | 25.43 |
| NSCLC4 | 28.29 | 24.68 | 30.95 | 30.24 | 29.86 | 28.48 | 28.80 |
| NSCLC5 | 29.78 | 28.70 | 33.78 | 33.43 | 32.86 | 33.06 | 32.83 |
| NSCLC6 | 29.63 | 26.11 | 31.15 | 30.55 | 29.36 | 29.36 | 29.13 |
| HBC1 | 25.26 | 24.48 | 26.77 | 27.14 | 31.37 | 25.61 | 26.73 |
| HBC2 | 27.02 | 24.98 | 28.79 | 31.69 | 31.44 | 29.31 | 27.07 |
| HBC3 | 24.92 | 25.35 | 27.04 | 30.33 | 28.14 | 28.26 | 26.07 |
| HBC4 | 26.49 | 25.68 | 29.99 | 28.92 | 27.60 | 26.99 | 28.94 |
| HBC5 | 24.80 | 25.19 | 28.94 | 27.65 | 26.68 | 26.60 | 26.36 |
| HBC6 | 28.58 | 26.68 | 32.00 | 31.72 | 31.00 | 30.12 | 30.15 |
| HC1 | 24.12 | 23.11 | 27.72 | 29.44 | 26.69 | 27.09 | 27.09 |
| HC2 | 26.27 | 24.36 | 30.00 | 32.40 | 28.64 | 27.44 | 27.59 |
| HC3 | 24.52 | 27.02 | 28.62 | 29.29 | 31.79 | 30.50 | 27.97 |
| HC4 | 29.78 | 24.06 | 30.05 | 29.46 | 28.39 | 27.27 | 28.06 |
| HC5 | 25.25 | 24.50 | 27.70 | 29.37 | 28.95 | 27.86 | 25.90 |
| HC6 | 31.70 | 25.74 | 31.99 | 31.15 | 30.79 | 28.53 | 30.34 |
| HC7 | 26.36 | 25.51 | 28.23 | 34.61 | 29.22 | 28.05 | 26.00 |
| CRC1 | 26.24 | 26.54 | 28.98 | 29.18 | 30.03 | 27.42 | 28.13 |
| CRC2 | 26.24 | 24.62 | 28.29 | 29.89 | 29.13 | 28.19 | 29.47 |
| CRC3 | 26.24 | 23.32 | 28.36 | 31.69 | 28.48 | 28.43 | 30.41 |
| CRC4 | 28.13 | 26.19 | 31.33 | 31.11 | 29.48 | 29.38 | 30.11 |
| CRC5 | 30.51 | 28.50 | 31.19 | 33.48 | 32.40 | 31.53 | 31.73 |
| CRC6 | 26.55 | 23.93 | 28.62 | 27.08 | 26.54 | 25.30 | 26.13 |
| BrCa1 | 26.91 | 25.16 | 28.09 | 28.44 | 32.43 | 26.16 | 25.95 |
| BrCa2 | 25.07 | 23.98 | 27.25 | 30.00 | 28.71 | 28.02 | 24.70 |
| BrCa3 | 27.71 | 26.41 | 28.13 | 31.58 | 30.60 | 30.11 | 27.38 |
| BrCa4 | 30.91 | 26.51 | 34.65 | 34.69 | 33.18 | 32.47 | 32.23 |
| BrCa5 | 27.12 | 25.19 | 32.03 | 30.01 | 29.11 | 28.60 | 28.93 |
| BrCa6 | 26.22 | 23.88 | 32.71 | 29.03 | 27.64 | 27.85 | 28.58 |

# NSCLC: Non-small cell lung carcinoma, CRC: Colorectal cancer, HBC: Hepatobiliary cancer, BrCa: Breast cancer, HC: Healthy subjects.

# Supplementary Table3. The baseline characteristics of the seven reference genes Ct values.

|  | Total | | NSCLC | | HBC | | HC | | CRC | | BrCa | |
| --- | --- | --- | --- | --- | --- | --- | --- | --- | --- | --- | --- | --- |
|  | Mean | SD | Mean | SD | Mean | SD | Mean | SD | Mean | SD | Mean | SD |
| ACTB | 27.05 | 2 | 27.58 | 1.95 | 26.18 | 1.48 | 26.86 | 2.83 | 27.32 | 1.73 | 27.32 | 1.98 |
| B2M | 25.4 | 1.33 | 26.1 | 1.42 | 25.39 | 0.75 | 24.9 | 1.29 | 25.52 | 1.92 | 25.19 | 1.13 |
| GAPDH | 29.52 | 2.14 | 29.63 | 2.8 | 28.92 | 1.94 | 29.19 | 1.58 | 29.46 | 1.41 | 30.48 | 3.05 |
| GNAS | 30.49 | 1.96 | 30.97 | 1.6 | 29.57 | 1.98 | 30.82 | 2.05 | 30.41 | 2.21 | 30.63 | 2.26 |
| OAZ1 | 29.63 | 1.91 | 30 | 2.12 | 29.37 | 2.14 | 29.21 | 1.66 | 29.34 | 1.92 | 30.28 | 2.19 |
| PTMA | 28.52 | 1.79 | 29.49 | 1.88 | 27.82 | 1.72 | 28.1 | 1.16 | 28.38 | 2.07 | 28.87 | 2.18 |
| YWHAZ | 28.16 | 2.06 | 28.47 | 2.58 | 27.55 | 1.62 | 27.56 | 1.5 | 29.33 | 1.96 | 27.96 | 2.63 |

# NSCLC: Non-small cell lung carcinoma, CRC: Colorectal cancer, HBC: Hepatobiliary cancer, BrCa: Breast cancer, HC: Healthy subjects.

# Supplementary Table4. The Ct values of the three reference genes in the 50 subjects.

|  | ACTB | B2M | GAPDH |
| --- | --- | --- | --- |
| NSCLC1 | 20.36 | 20.18 | 23.96 |
| NSCLC2 | 23.47 | 23.98 | 27.06 |
| NSCLC3 | 18.26 | 20.08 | 24.03 |
| NSCLC4 | 20.32 | 26.70 | 27.21 |
| NSCLC5 | 16.84 | 23.01 | 22.30 |
| NSCLC6 | 23.30 | 24.16 | 26.58 |
| NSCLC7 | 25.02 | 24.25 | 26.03 |
| NSCLC8 | 24.03 | 20.75 | 24.15 |
| NSCLC9 | 24.98 | 22.76 | 26.18 |
| NSCLC10 | 19.95 | 19.99 | 23.75 |
| HBC1 | 19.33 | 21.16 | 23.17 |
| HBC2 | 21.07 | 21.01 | 23.45 |
| HBC3 | 20.80 | 23.27 | 23.51 |
| HBC4 | 19.18 | 20.43 | 22.17 |
| HBC5 | 18.32 | 27.27 | 27.27 |
| HBC6 | 19.06 | 33.06 | 29.64 |
| HBC7 | 24.30 | 25.45 | 27.87 |
| HBC8 | 20.27 | 20.07 | 24.74 |
| HBC9 | 21.31 | 21.61 | 27.83 |
| HBC10 | 22.45 | 22.73 | 26.29 |
| HC1 | 20.23 | 25.64 | 23.86 |
| HC2 | 22.18 | 26.42 | 25.76 |
| HC3 | 18.24 | 25.44 | 27.74 |
| HC4 | 21.72 | 23.18 | 25.05 |
| HC5 | 22.38 | 24.42 | 26.11 |
| HC6 | 25.10 | 29.05 | 28.52 |
| HC7 | 24.99 | 25.16 | 28.46 |
| HC8 | 21.47 | 25.80 | 25.46 |
| HC9 | 25.60 | 20.26 | 27.03 |
| HC10 | 21.59 | 20.62 | 24.75 |
| CRC1 | 19.45 | 20.50 | 23.27 |
| CRC2 | 20.97 | 21.90 | 24.73 |
| CRC3 | 21.32 | 20.76 | 24.68 |
| CRC4 | 25.81 | 25.25 | 25.25 |
| CRC5 | 22.70 | 20.93 | 26.38 |
| CRC6 | 21.50 | 20.31 | 24.71 |
| CRC7 | 23.16 | 26.03 | 25.61 |
| CRC8 | 22.57 | 21.32 | 23.21 |
| CRC9 | 20.27 | 22.21 | 23.68 |
| CRC10 | 24.04 | 22.44 | 26.83 |
| BrCa1 | 24.01 | 24.94 | 27.78 |
| BrCa2 | 21.95 | 22.50 | 28.27 |
| BrCa3 | 22.08 | 23.37 | 25.28 |
| BrCa4 | 20.99 | 21.37 | 23.62 |
| BrCa5 | 19.87 | 19.83 | 24.02 |
| BrCa6 | 22.43 | 24.44 | 27.75 |
| BrCa7 | 20.41 | 28.88 | 27.89 |
| BrCa8 | 25.77 | 25.58 | 27.05 |
| BrCa9 | 24.88 | 23.76 | 25.67 |
| BrCa10 | 24.20 | 21.67 | 25.68 |

# NSCLC: Non-small cell lung carcinoma, CRC: Colorectal cancer, HBC: Hepatobiliary cancer, BrCa: Breast cancer, HC: Healthy subjects.

Supplementary Table5.Differential expression of GAPDH in diseases

| **Ensembl Id** | **Symbol** | **Disease** | **Control mean** | **Case mean** | **Log2FC** | **FDR** | **P-value** |
| --- | --- | --- | --- | --- | --- | --- | --- |
| ENSG00000111640 | GAPDH | ST-segment elevation myocardial infarction | 6.08 | 9.03 | 2.95 | 0 | 0 |
| ENSG00000111640 | GAPDH | HIV, Dengue, H1N1 | 8.18 | 9.23 | 1.06 | 0.4 | 0.03 |

Supplementary Table 6.Verification of the relative expression levels of FLNA gene

|  | **LC** | **HC** | **P** |
| --- | --- | --- | --- |
| **FLNA** |  |  |  |
| (Mean ± SD)for 2^-△△Ct^ | 2.001±0.887 | 1.000±0.5815 | 0.0002 |
